# Complex Nasal and Periorbital Reconstruction Using Locoregional Flaps: A Case Report 

**Published:** 2012-07

**Authors:** Nikhil Panse, Parag Sahasrabudhe, Rajendra Dhondge

**Affiliations:** Department of Plastic Surgery, BJ Medical College and Sassoon Hospital, Pune, India

**Keywords:** Facial trauma, Forehead, Temporoparietal, Flap, Eyebrow, Nose, Reconstruction

## Abstract

Facial reconstruction is one of the most challenging problems faced by a reconstructive surgeon. We present a case of complex facial reconstruction with a composite trauma to the nose resulting in near total loss of skin and lining along with complete loss of left eyebrow with exposed frontal bone and partial loss of the left eyelid. We combined a temporoparietal fascial flap for reconstruction of the eyebrows and covering the exposed frontal bone and prefabricated forehead flap with skin graft for nasal reconstruction. Proper planning and staging of the surgical procedures and use of local flaps gave us good aesthetic and functional outcome with a satisfied patient.

## INTRODUCTION

Facial reconstruction has always been challenging to the reconstructive surgeon. When the reconstruction of the face involves two or more units of the face with trauma to the adjacent tissue, the reconstruction becomes even more difficult and challenging.^1^ Our patient had a composite defect involving the nose and periorbital region. Goals of reconstruction included restoration of a functional nasal airway and redefinition of the contours of the nose as well as its relationship to the cheek and lip; restoration of an aesthetic eyebrow and functioning ocular unit with the least amount of morbidity to the patient. This article details a multistage approach to repairing such a defect using an prefabricated forehead flap for nasal reconstruction and temporoparietal fascial flap for eyebrow reconstruction.

## CASE REPORT

**Fig. 1:**
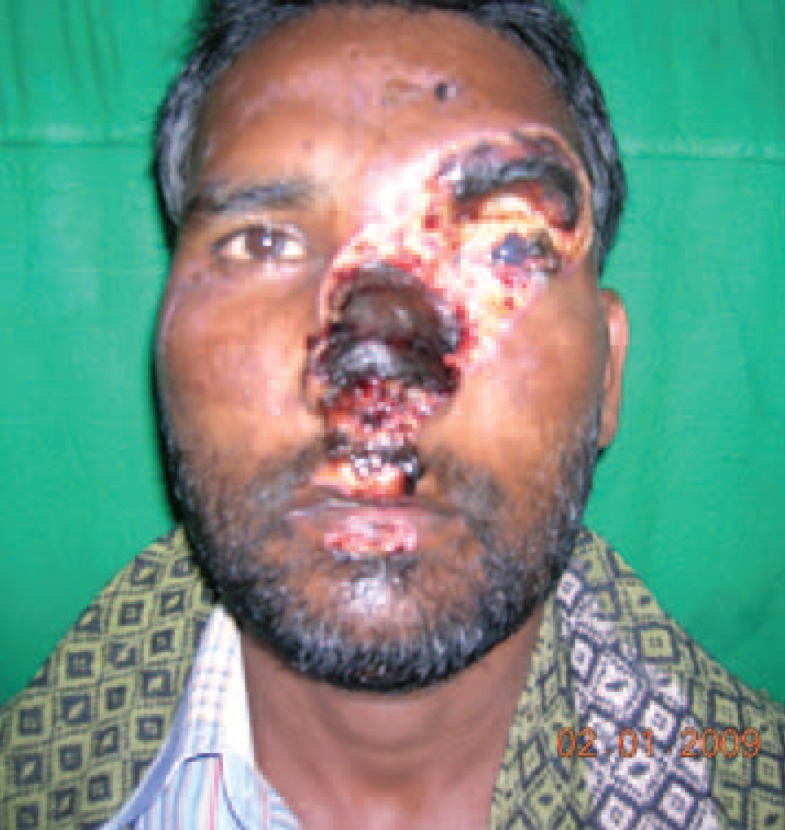
Pre debridement picture of patient.

**Fig. 2:**
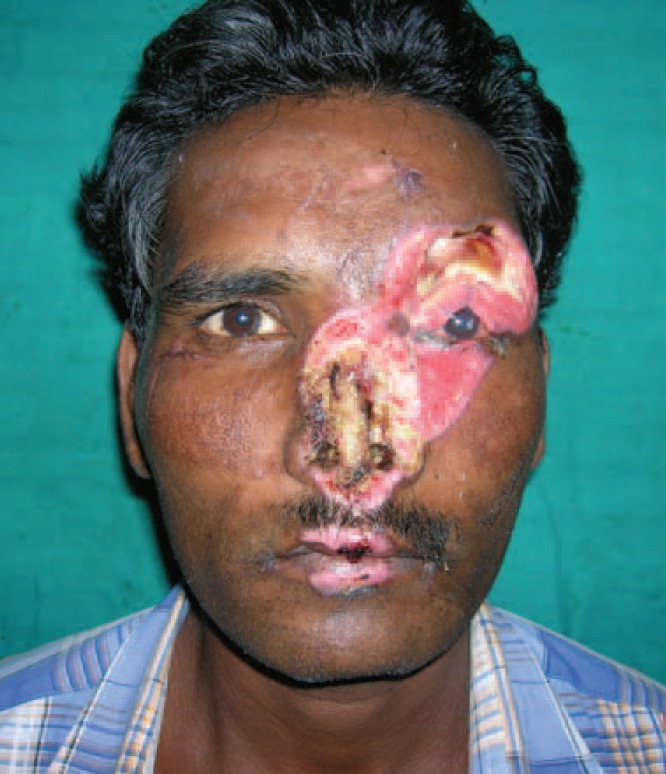
Post debridement picture of patient.

**Fig. 3:**
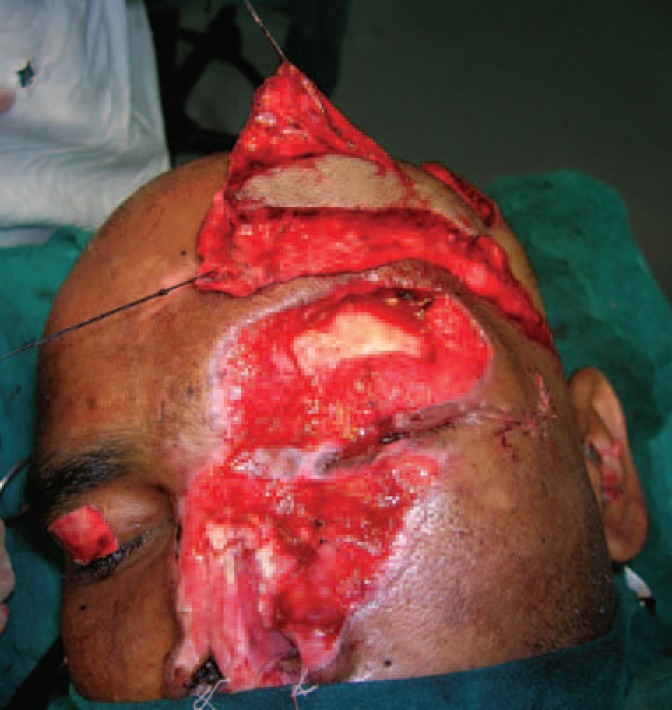
Harvested TP fascia.

A 30 years old male patient came with history of road traffic accident before 15 days. There was a full thickness eschar formed over the nose, and the left supraorbital region and loss of around forty percent of the upper eyelid on the left side leading to exposure keratitis (Figures 1-3).

X ray showed no fracture. The left eye had a patch of opacity with blurred vision on the left side (Figure 1). Post debridement, the defect comprised of near total loss of skin and lining of the nose, total loss of left eyebrow, around 40% loss of the upper eyelid and exposed frontal bone in the left supraorbital region (Figure 2). Patient was posted for definitive surgery within the next few days. The reconstructive challenges were eyelid reconstruction, reconstruction of the nose, coverage of the exposed bone of the supraorbital region and eyebrow reconstruction. The schema of the procedure and various stages is described in Table 1.

**Table 1:** The schematics of various steps in reconstruction

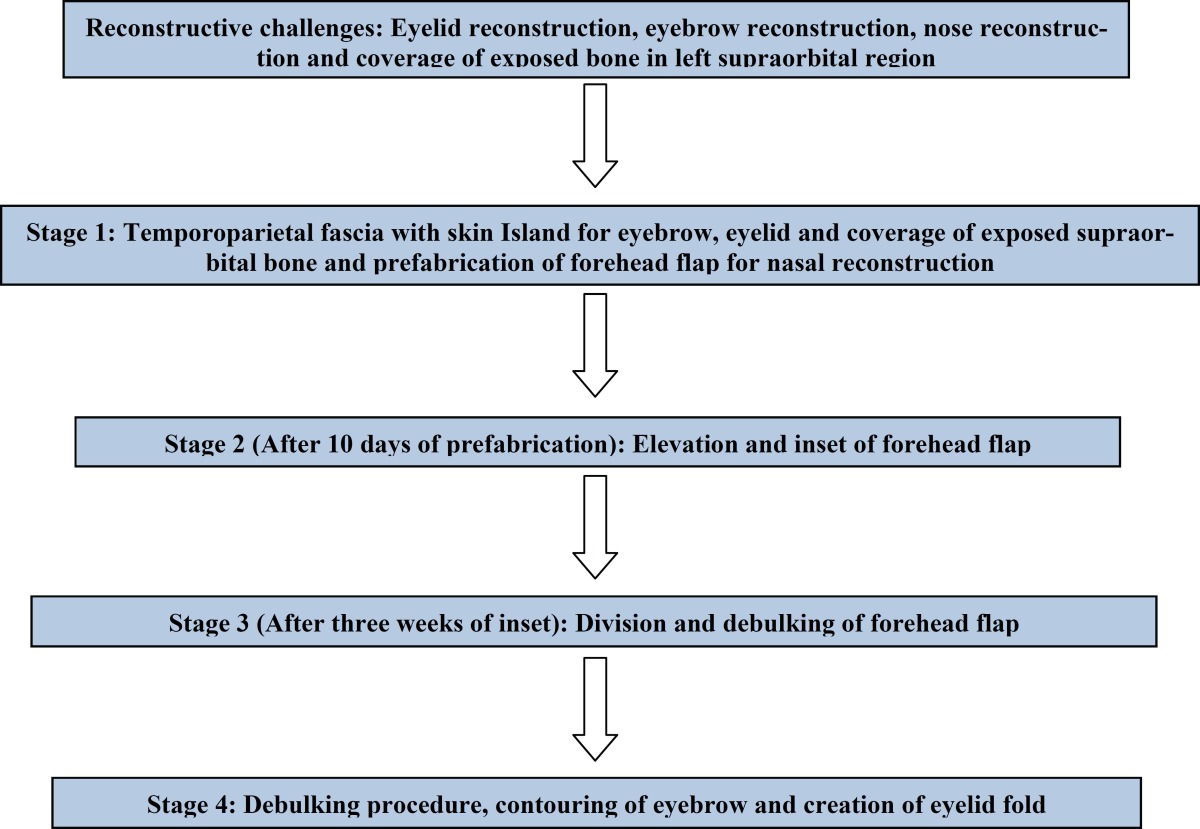

An internal nasal splint was kept to maintain pressure over the grafted area for prolonged period of over six months.

**Fig. 4:**
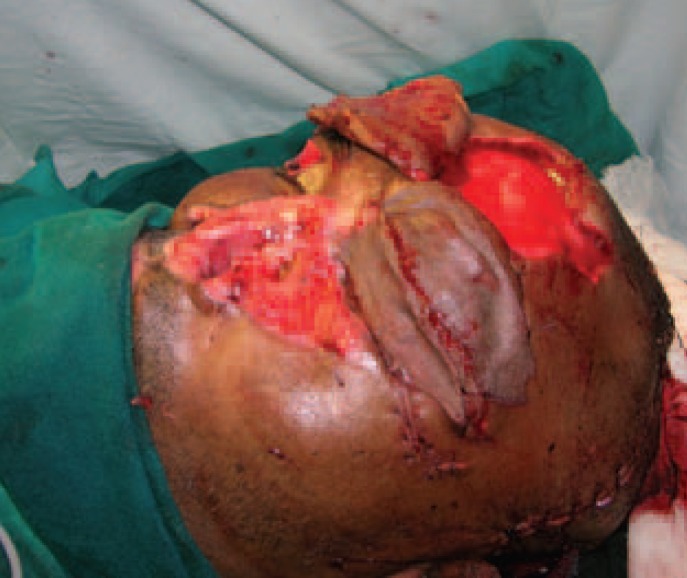
Prefabricated forehead flap.

**Fig. 5:**
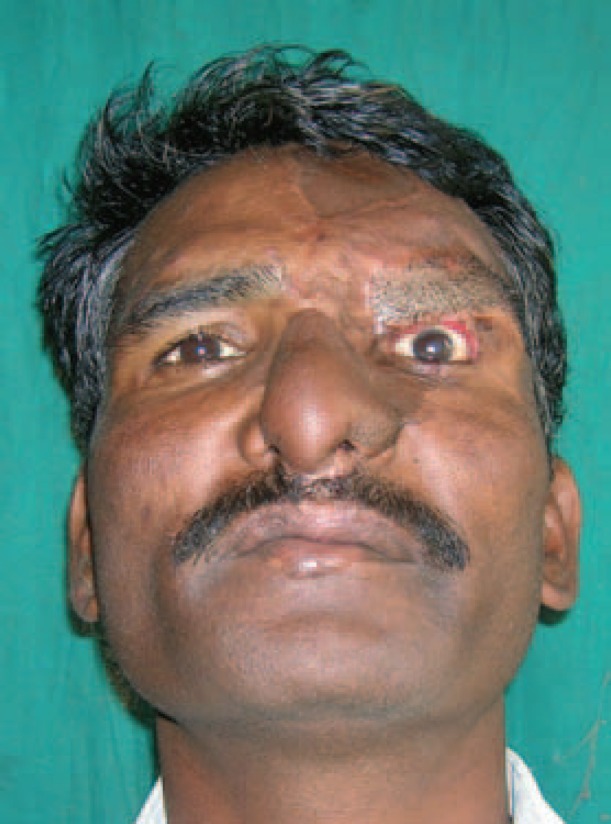
Post op at 12 weeks follow up.

**Fig. 6:**
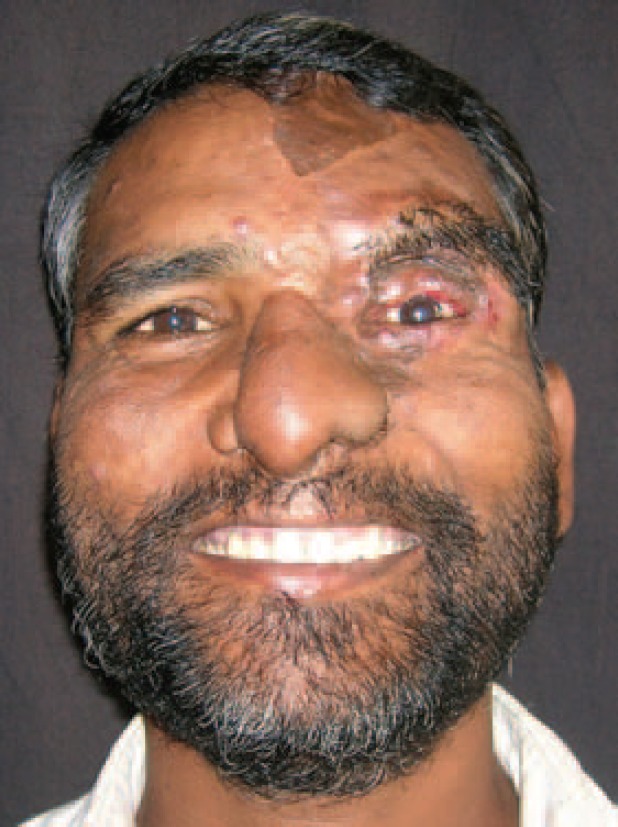
Post op at 20 weeks follow up.

At 20 months follow up, the patient had no functional problems. Both the nasal apertures were patent and there was no blockage. There were no visual complaints like blurring and there were no corneal opacities. The pictures at various stages were shown (Figures 3-6). Although the patient had no functional problems, we felt that the aesthetic outcome was suboptimal, and suggested further procedures like debulking, dorsal augmentation and tip enhancement. However, the patient was satisfied with the outcome and not desirous of other procedures suggested to him.

## DISCUSSION

Defects of complex aesthetic subunits of nose and eyebrow and limited availability of donor area contribute to the difficulties encountered in achieving a good aesthetic functional outcome in complex facial reconstructions. Because of its ideal color and texture, forehead skin is acknowledged as the best donor site with which to resurface the nose.^1^ Surgical treatment is extremely difficult with the combined defects of skin, cartilage, and nasal mucosa. Besides, efforts geared toward ascertaining the best aesthetic outcome, an important concern is restoring normal nasal function. The prefabricated forehead flap has been used to provide an anatomically sufficient amount of nasal skin and nasal mucosa for whole-layer wide nasal defects in only three sessions without necessitating an additional flap.^2^

Lining for full-thickness alar or unilateral tip defects that had a vertical dimension (caudal to cephalic) of 1.0 cm or less can be provided using the thin skin lining the remaining nasal vestibule.^3^ Defects with vertical dimensions as large as 1.5 cm may sometimes be lined using this method if the remaining skin of the interior of the lower nasal vault is of sufficient size.^3^ Bipedicle vestibular skin advancement flaps are insufficient to line fullthickness defects of the unilateral tip or ala that measure more than 1.5 cm in vertical height. There was insufficient skin between the defect margin and the necessary intercartilaginous incision made at the junction of the upper lateral cartilage and the alar cartilage. In these circumstances, an ipsilateral septal mucoperichondrial flap hinged on the caudal border of the cartilaginous septum can provide adequate mucosa to reline the entire interior of the ala and nasal dome.^3^ Basing the flap on the entire vertical height of the caudal septum rather than on a narrower, 1.5-cm-wide pedicle adjacent to the nasal spine as advocated by Burget and Menick^4^ served to support the flap and stabilize the pedicle, thus preventing torsion that may compromise the vascular supply to the flap. However, construction of the pedicle of the flap in this manner requires that the flap spans the distance from the caudal septum to the lateral aspect of the lining defect. This means the flap will, in part or completely, obstruct the nasal passage until it is detached from the septum. Considering this flap as an option for bilateral nasal lining means that total nasal blockage for a period of up to 3 weeks, which would mean significant discomfort to the patient. A muco-perichondrium hinge flap and septal composite chondromucosal pivotal flap have also been described for nasal lining reconstruction, but for limited mucosal defects.5 The middle and inferior turbinates have been described to line limited mucosal defects of the nose. These turbinates are richly supplied by a vascular network arising from a lateral descending branch of the sphenopalatine artery.^5^

Burget *et al. *reported that microvascular free flaps have proved to be highly reliable and efficacious for restoration of missing elements of the nasal lining and adjacent facial soft-tissue defects in total and subtotal nasal reconstruction.^6^ However, it needs a great deal of technical expertise and facilities that are not universally available.

In our patient, there was a composite defect of the skin, cartilage and lining and the tissue requirement was large. In an acute setting of trauma, expanding the forehead would be time consuming. There was open wound with exposed bone over the left supraorbital region which would increase the chances of infection in case that an expander was used. Other options for lining like the bipedicle vestibular skin flaps, septal mucoperichondrial flaps and turbinate flaps were not an option considering the enormously large mucosal defect and smaller donor area. We therefore, felt that lining for mucosa with a prefabricated flap by skin grafting was a better and considered a prefabricated forehead flap for nasal reconstruction.

Menick in relation to free tissue transfers stated that "Distant skin always appears as a mismatched patch within residual normal facial skin."^1^ In addition, earlier techniques using a single large nasal lining flap or bilateral nasal lining vaults incurred a high incidence of airway obstruction.^1^

In our patient, in spite of the shrinkage of the graft, which we tried to limit by keeping internal nasal splints, there was no nasal airway obstruction, and the nasal apertures were patent at 20 months follow-up. The eyebrow was an important subunit of facial aesthetics and expression. Partial or total absence of the eyebrow was an unacceptable and disturbing condition. Reconstruction of cutaneous eyebrow defects is a challenge, as eyebrow positioning provides an important role in communication, cosmesis, and signaling age, gender, and emotional status. Due care must be taken to maintain eyebrow symmetry and to avoid distortion of the hairline. There are several options available for reconstruction of the eyebrow. Each method has advantages and disadvantages. The selection must be individualized, depending on the extent and location of the eyebrow defect in relationship to other structures, gender, and age of patients. Understanding the unique anatomy and function of the eyebrow, including its movement in facial expression, is useful in achieving good reconstructive outcomes while maintaining normal eyebrow function.^7^

Motamed and Davami believed that composite graft was preferable for females while the superficial temporal artery island flap seemed more suitable for males.^8^

Stamatopoulos *et al. *observed that the temporal vessels enjoy a constant anatomical course, along with a large diameter and long axis, and excellent results can be achieved in eyebrow reconstruction through applying methods of much more simplicity with reliability.^9^ Cheney *et al. *described 21 cases using the flap for a variety of reconstructions in the head and neck including eyebrow reconstruction.^10^ Bozkurt *et al. *reported a case where, a superficial temporal fascial flap was designed for reconstruction of the eyebrow, upper and lower eyelids, and lacrimal drainage system in a onestage procedure in facial burn patient.^11^

Our patient had a complete eyebrow loss and exposed frontal bone. Other options of eyebrow reconstruction commonly used like composite grafts and subcutaneous island flaps were not considered as an option because of the large tissue requirements and the exposed frontal bone. We therefore, planned for the temperoparietal fascial flap based on the posterior branch of the superficial temporal vessel with ample amount of fascia. Alopecia of the suture lines was a known and described complication after harvestation of this flap. However, we did not encounter this complication because of meticulous elevation of the scalp flaps in the proper plane immediately deep to the hair follicles. The cauterization of the skin edges was also kept to minimal to further reduce chances of suture line alopecia. The only drawback of this procedure was the growth of the scalp hair on the eyebrow region which necessitated recurrent trimming on the part of the patient. Proper planning and staging of the surgical procedures with use of locoregional flaps gave us good aesthetic and functional outcome with a satisfied patient.

## CONFLICT OF INTEREST

The authors declare no conflict of interest.
